# Food Additive (Sodium benzoate)-induced Damage on Renal Function and Glomerular Cells in Rats; Modulating Effect of Aqueous Extract of *Atriplex halimus* L.

**DOI:** 10.22037/ijpr.2020.111634.13272

**Published:** 2021

**Authors:** Khaoula Zeghib, Djahra Ali Boutlelis

**Affiliations:** a *Department of Chemistry, Faculty of Exact Sciences, University of El-Oued, El-Oued 39000, El-Oued, Algeria. *; b *University of El-Oued, VTRS Laboratory, Fac. Technology, 39000 El-Oued, Algeria. *; c *Department of Biology, Faculty of Natural Sciences and Life, University of El-Oued, El-Oued 39000, Algeria.*

**Keywords:** Antioxidants, Kidney, Oxidative stress, Rats, Sodium Benzoate, Toxicity

## Abstract

The aim of the current study was to investigate the preventive and curative effect of *Atriplex halimus* L. (Ah) extract against the kidney damages induced by Sodium benzoate (SB) in rats. Thirty male albino rats were divided into five groups of 6 rats each: Control, Ah, SB, AhP+SB and SB+AhC. SB (100 mg/kg b.w) was added to drinking water for 15 weeks. Aqueous extract of aerial parts of *Atriplex halimus* received intragastrically during the last 30 days of SB exposure for curative treatment (AhC) and all the duration of SB exposure for preventive treatment (AhP). Some Biochemical markers, oxidative stress parameters and histopathology of kidney tissue were studied. Administration of Sodium benzoate to rats caused a loss of weight and a significant elevation in creatinine, urea, renal malondialdehyde levels and lactate dehydrogenase activity. These changes were accompanied by decreasing in antioxidant defenses, like reduced glutathione level, catalase and glutathione S transferase activities in the kidney. Histopathological studies showed a massive degeneration in kidney tissue in SB-exposed rats. However, treatment with *Atriplex halimus* (*A. halimus*) restored the altered of biochemical and oxidative stress markers. *A. halimus* also regenerated the architectural kidneys lesions to near control. With more protection offered in the curative than preventive models of treatment. In conclusion, the results demonstrate that Sodium benzoate damages kidney structure and function and is a nephrotoxic substance. *Atriplex halimus* was able to improve the renal damage as an antioxidant and a nephroprotective agent.

## Introduction

In the last years, with the excessive production and consumption of processed and convenience foods, the use of food additives has increased enormously (1). These additives are widely used for various purposes and the majority of them are preservatives (2). Food preservatives are substances added to prevent food deterioration from microorganisms, enzymes, and oxygen exposure (3). Sodium benzoate (SB) is one of the common food preservatives (4), known sodium salt of benzoic acid with the chemical formula C_7_H_5_NaO_2_ and number E211 (5). It is characterized by good stability and excellent solubility in water, which led to wide use in several food products such as margarine, sauce, marmalade, gelatin, liqueurs, beer, fruit juice, and soft drinks (6). According to the Food and Drug Administration (FDA), SB is commonly considered as safe (GRAS) with the allowed limit level in food 0.1% (1000 ppm) (7). Further, the International Program on Chemical Safety (IPCS) reported no adverse health effects in humans at doses of 647-825 mg/kg of body weight per day (8). However, many studies report that the intake of sodium benzoate provokes urticaria, angioedema, asthma, childhood hyperactivity, anxiety and it has also been induced DNA damage (8, 9). 

The kidneys have a special role in eliminating toxic chemicals from the body by concentrating them within its tubules and excreting them into the urine (10). These functions render it susceptible to damage as the result of prolonged cumulative exposure to toxic metabolites compounds (11). SB is further cleared by the kidneys and their continuous use may cause adverse effects like any other chemicals (12). In fact, it has been assumed that the generation of oxidative stress in the kidney is the essential mechanisms of xenobiotics induced nephrotoxicity. Therefore, a natural diet containing antioxidant compounds is important for preventing or reducing the toxic effects of chemicals in the kidney (13). Plants have extraordinary therapeutic virtues. Their uses for the treatment of several diseases in living beings are very old (14).

*Atriplex halimus* L. is a halophytic shrub of semi-arid and arid Mediterranean areas belonging to the Chenopodiaceae subfamily, commonly known as *Guetaf* (15-18). This plant has a good nutritive and energetic value, not only for livestock but also as food for nomads and the local steppe population. Then, in many regions in Algeria and Tunisia, people consumed *Guetaf* by preparing them like spinach (19). In traditional medicine, *A. halimus *has been used to treat heart disease, diabetes, syphilis, rheumatism (20), anemia (18), and chest ailments and to cure stomach and intestinal worms (16, 17 and 21). *A. halimus* is also used to treat urinary tract inflammation (cystitis) and urinary lithiasis, drainage of skin and kidney, diuretic and depurative; it accompanies any diet that requires tissue drainage and desincrustation of wastes and toxins (19). Moreover, the previous study indicated that* A. halimus *contains a wide range of bioactive metabolites like flavonoids, tannins, saponins, alkaloids and resins (22, 23), and it is a source of vitamins A, C and D (21). These molecules are well known for showing therapeutic activity as well as exhibiting physiological activity (22). Given these considerations, this paper proposes a new approach to studying the potential toxicity of food preservatives (sodium benzoate) in the kidneys, and to determine nephroprotective effects of *Atriplex halimus* against SB in rats. 

## Experimental

*Chemicals and reagents*

All chemicals were of analytical grade and purchased from Sigma-Aldrich, Mo, USA.

*Collection, identification, and extraction of plant material*

*Atriplex halimus* L. aerial parts were collected from a village of Biskra state, Algeria and then identified by a botanist at the herbarium in the department of biology, University of El-Oued, Algeria. The samples were washed, dried at room temperature, and pulverized for extraction by the maceration method. Ten grams of the aerial parts of Ah powder was mixed with 100 mL distilled water at room temperature in the dark for 24 h. Then, the mixture was filtered through Whatman filter paper and then the filtrate was concentrated using a rotary evaporator and incubated at 40 °C to dry completely (24). The percentage yield of the aqueous extract is 19.29 ± 0.24% of the dried sample (w/w). 

*Phytochemical Screening*

Classical methods were used to identify the phytochemicals provides in the extracts of flavonoids (25), saponins, cardiac glycosides (26), terpenoids, mucilage (27), alkaloids (28), anthocyanins (29), tannins (30), coumarins, and carotenoids (31).

*Estimation of Total Phenolic*

Total phenolic content was determined using the Folin-Ciocalteu method (32). Two tenth milliliters of the aqueous extract of *Atriplex halimus* was mixed with 1 mL of Folin-Ciocalteu reagent (diluted 1:10) and 0.8 mL of saturated sodium carbonate (75 g/L). After 2 h of reaction at room temperature in the dark, the absorbance at 765 nm was calculated. The tests were performed three times in order to guarantee the reproducibility of the results. The total phenolic content was expressed in mg equivalent of Gallic Acid (GAE) per g of sample.

*Estimation of Total Flavonoids*

The total flavonoid content of *A. halimus* extract was estimated by the aluminum chloride colorimetric method (33). One and half milliliters of a 2% AlCl_3_ solution was added to 1.5 mL of sample or standard. After incubation for 30 min at room temperature, the absorbance was measured at 430 nm. Quercetin (Q) was used as a standard for plotting the calibration curve. The tests were carried out three times in order to ensure the reproducibility of the results. The results were represented in mg of Quercetin equivalent per g of sample.

*In-vivo study*

*Animals and Handling*

**Figure 1:**
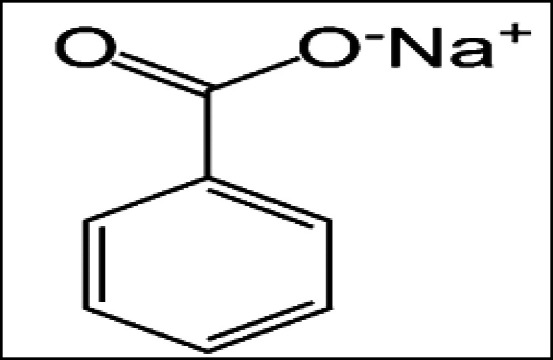
Chemical structure of sodium benzoate (1).

Thirty male Wistar albino rats, weighing 223.72 ± 5.37 g, were obtained from the animal house of the Pasteur Institute, Algeria. They were placed in animal’s house of the molecular and cellular biology department, the university of El-Oued, Algeria, in controlled (19 ± 1 °C) temperature, photoperiod (12 h light/12 h dark) cycle, and relative (64 ± 2%) humidity. Standard rat food and tap water were available ad libitum for the duration of the experiment. The rats were adapted for two weeks before tests under the same laboratory conditions. The experimental procedures were performed according to the National Institute of Health Guidelines for Animal Care and approved by the Ethics Committee of our Institution. The animals were randomly divided into five groups, each containing 6 rats as follow: 

Groups 1: was serving as a control and received normal water; 

Groups 2: was treated orally by gavage 200 mg/kg, b.w (3 days/week) aqueous extracts of *Atriplex halimus* (Ah) for 15 weeks;

Groups 3: was received in drinking water 100 mg/kg, b.w/day of sodium benzoate (SB) daily for 15 weeks (Figure 1);

Groups 4: was firstly treated with SB for 11 weeks and then treated curatively by 300 mg/kg, b.w/day of Ah (SB+AhC) for 30 days; Groups 5: was concomitantly administered Ah (200 mg/kg, 3 days/week) preventively with SB for 15 weeks (AhP+SB);

During treatment, body weight was recorded periodically during the experiment weeks.

*Blood collection and preparation of tissue samples*

At the end of the experiment, rats fasted for 16 h, sacrificed after anesthetized with chloroform by inhalation, and blood samples were collected. The serum was prepared by centrifugation and utilized for creatinine, urea, uric acid, albumin, and total protein concentrations and lactate dehydrogenase (LDH) activity assays. Blood glucose was measured by glucometer. The kidney was excised, rinsed, and weighed. Part of the tissue was stored at -20 °C for homogenate preparation. Another part of the kidney was transferred to fixative for histopathological examination.

*Measurement of biochemical parameters*

Serum creatinine, urea, uric acid, albumin, and total protein concentrations and LDH activity were measured using commercial kits obtained from Spinreact (Barcelona, Spain).

*Antioxidants measurement*

*Preparation of homogenates (1)*

Tissue kidney homogenates were prepared at 10% (w/v) in Tris buffer saline (Tris 50 mM, NaCl 150 mM, pH 7.4). The homogenate was centrifuged at 4000 revolution/min for 30 min at 4 °C. The supernatant was separated and used for the determination of oxidative stress markers.

*Determination of Malondialdehyde (MDA) level (2)*

MDA level was assayed according to the method described by Quintanilha *et al*. (1982) (34). 2 mL of MDA reagent (15% (w/v), trichloroacetic acid, 0.375% (w/v) thiobarbituric acid, and 0.25N hydrochloric acid) was added to aliquots of 1 mL diluted homogenate mixed with 20 µL of 2% (w/v) ethanolic solution of butylated hydroxytoluene. After incubating the mixture for 15 min in a boiling water bath and cooling in ice-cold, the precipitate was recuperated by centrifugation, and the absorbance was measured at 532 nm. MDA level was expressed as nmol of MDA/mg protein. 

*Determination of Reduced glutathione (GSH) level (4)*

 GSH was measured by the method of Ellman (1959) using DTNB (5,5′-dithiobis-(2-nitrobenzoic acid) reagent. 0.8 mL of kidney homogenate was mixed with 0.2 mL of 0.25% sulphosalicylic acid and the mixture was centrifuged at 1000 rpm for 10 min. Then, 0.5 mL of supernatant was added to 1 mL TBS (pH 7.4) and 0.025 mL (0.01 M) DTNB. The absorbance was recorded at 412 nm. The results were expressed as nmol GSH/g tissue (35).

*Determination of Glutathione-S-transferase (GST) activity (5)*

GST activity was performed using the method of Habig *et al.* (1974) (36). To 2.8 mL phosphate buffer (pH 6.5), 25µL of kidney homogenates and 100 µL of (30 mM) GSH were added and the reaction was initiated by the addition of 100 µL (10 mM) CDNB (1-chloro, 2,4-dinitrobenzene). The absorbance was detected at 340 nm. GST activity was calculated in terms of μmol CDNB-GSH conjugate formed/min/g tissue.

*Assay of Catalase (CAT) activity (6)*

CAT activity was estimated according to Aebi (1984) method. The reaction was started by adding 200 μL of H_2_O_2_ (0.030 M) to 20 μL of supernatant and 780 μL of phosphate buffer (KH_2_PO_4_, 0.1 M, pH 7.5). The decomposition of H_2_O_2_ was monitored by following the decrease in absorbance at 240 nm every 30 sec for 2 min. The enzymatic activity was expressed in terms of international unit per minute and per gram of tissue (IU/min/g of tissue) (37).

*Protein level assay (7)*

The protein content of kidney homogenates was determined as described by the Bradford method, and used bovine serum albumin as the standard (38).

*Histopathological study*

Part of kidney tissues of all experimental groups was immersed in fixative (10% formaldehyde solution) until the time of slice preparation. It was dehydrated in ascending graded concentrations of ethanol, cleaned with toluene, embedded in paraffin and sliced into 5 μm thick sections by a rotary microtome. Then, it was stained with hematoxylin and eosin. Histopathological evaluation was performed under a light microscope. 

*Statistical Analysis*

Data were expressed as mean ± standard deviation (SEM) of six animals. Statistical analysis was carried out by using the Student *t*-test to compare means among the groups. Differences were considered significant at *p *< 0.05.

## Results

*Phytochemical Screening*

Phytochemical analysis revealed the presence of saponins, tannins, flavonoids, alkaloids, coumarins, cardiac glycosides, anthocyanins and terpenoids in the extract of *Atriplex halimus* (Table 1).

*Phenolic and Flavonoids Compounds*

Total Phenolic and Flavonoids Compounds was expressed in terms of gallic acid equivalents (mg of GAE/g sample) and of Quercetin equivalents (mg of Q/g sample) respectively, using the following equation based on the calibration curve: Y = 0.0104x + 0.0819, R^2 ^= 0.9925 for phenolic compounds and Y = 0.0247x + 0.1164, R^2 ^= 0.9639 for flavonoids compounds. Total phenolic and flavonoids contents of *A. halimus* obtained from water solvent were represented in Table 2.

*Body and Relative kidney weight*

Sodium benzoate (as a food additive) supplement caused a decrease in body weight gain (*p* < 0.05) and no effect on relative kidney weight compared to the control rats. No effect was observed on body weight gain and relative kidney weight of *Atriplex halimus* alone treatment in rats when compared to the control. Preventive *A. halimus* treatment in SB rats was significantly (*p* < 0.01) increase body weight gain compared to SB rats, but no effect for curative treatment of extract aqueous of Ah compared to control (Table 3).

*Blood biochemical values*

As shown in Table 4, Sodium benzoate (SB) supplement caused a significant increase in serum creatinine (*p* < 0.01) and urea (*p* < 0.05) level and LDH activity and a significant decrease in blood ratio urea/creatinine (*p* < 0.01) and serum protein (*p* < 0.01) levels. Also, no effect was observed on Ah alone or SB supplement on blood glucose and serum albumin concentration when compared to control. Meanwhile, *A. halimus* made a recovery in the above mentioned biochemical parameters either for curative or preventive treatment. In another way, treatment with the aqueous extract of *Atriplex halimus* alone does not affect most of these parameters.

*Oxidative stress parameters*

As seen in Table 5, results shown a significant increase (*p < *0.05) in MDA level and a significant decrease in GSH concentration (*p < *0.05), GST (*p < *0.01) and CAT (*p < *0.05) activities in SB group compared to the corresponding control values. Treatment with *A. halimus* partially restored the levels of MDA (*p < *0.001), GSH (*p < *0.05), CAT (*p < *0.01) and GST (*p < *0.05) activities for curative effect and only the level of MDA (*p < *0.05) and GSH (*p < *0.05) for preventive effect. In addition, treatment with the plant extract alone does not cause any change in oxidative stress parameters.

*Correlations between analyzed parameters*

The result in Table 6 represents the correlation between serum kidney markers (creatinine, urea and ratio urea/creatinine) and kidney oxidative stress markers (MDA, GSH, GST and CAT) for experimental groups. Our results showed that there was a significant positive correlation between serum creatinine concentration and kidney MDA level (*p < *0.05) and a negative correlation with GSH level (*p < *0.01) and GST activity (*p < *0.05), while there is no significant correlation between urea concentration and kidney oxidative stress markers. In addition, there is a significant positive correlation between blood ratio urea/creatinine and antioxidant markers GSH (*p < *0.05), GST (*p < *0.05) and CAT (*p < *0.01), but there was no significant correlation between the rests of correlation test.

*Histopathological study*

**Figure 2:**
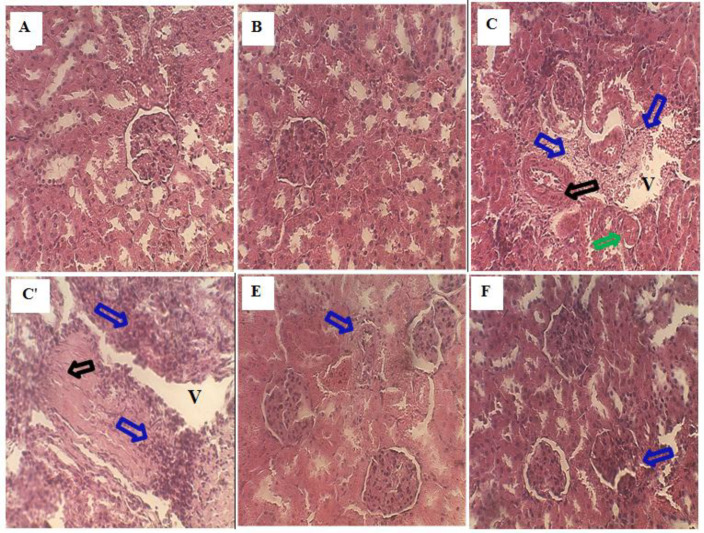
(A, ×40). The histological examination of the kidney from the control rat showed normal architecture. (B, ×40) The kidney section of *A. halimus* treated rats showed a healthy renal architecture. (C, C', ×40) Sodium benzoate treated rats' kidneys showed a Focus of inflammation (blue arrow), Glomerular necrosis (black arrow), Glomerular atrophy (green arrow) and vacuolation (V). Ah curative treated rat kidney showed a normal appearance of tubule and glomerulus and few inflammatory cellular infiltrations (blue arrow) (E, ×40). (F, ×40) Kidney sections of sodium benzoate rats prevented by *A. halimus* showed a moderate degree of kidney damage inflammatory cell, protection from tubule and glomerulus degradation

Microscopic observation of histological sections of the kidney from control and Ah treated rats showed normal renal parenchyma with normal glomeruli, tubules, and normal cortical and medullary areas (Figures 2A and 2B). However, histological sections of Sodium benzoate food additive treated rat kidney revealed massive degeneration represented by necrosis and glomerular atrophy apparently to their architecture with tubular necrosis, dilatation and vacuolation. In addition, hemorrhage, foci of inflammation and large space of Bowman, were observed in the kidney sections of this group (Figure 2C and 2C’). Kidney section of both (SB+AhC) and (AhP+SB) groups where rats received aqueous extract of *Atriplex* with SB of both curative and preventive treatment showed less inflammatory infiltrations as compared to treated rats with SB rats (Figures 2E and 2F). Histopathological changes are graded and summarized in Table 7. Histological grading was made according to four severity grades: 

− (none); + (mild); ++ (moderate) and +++ (severe).

## Discussion

Prolonged cumulative exposure to chemicals compounds could have adverse consequences on renal function and might be progressed to chronic kidney disease (11). On the other hand, it is generally reported that nutrition can attenuate the adverse effects of chemicals compounds (13). Therefore, this paper presents new research to evaluate the potential toxicity of sodium benzoate in kidney tissue for 105 days exposure in rats and to investigate the ameliorating effects of* Atriplex halimus* against SB toxicity.

The phytochemical investigation of *A. halimus* aqueous extract revealed the presence of a wide range of bioactive compounds which linked to a lower risk of most chronic and human degenerative diseases (39, 40). This finding was confirmed by Benhammou *et al*. (2009) (23) and Chikhi *et al*. (2014) (21). Further, quantitative analysis indicates that *A. halimus* extract contains a high content of total phenolic and flavonoids. These two constituents are strong antioxidants and good indicators for the antioxidant activity of this plant (41).

Decreased body weight is an important indicator for assessing the deterioration of health status (42). In our study, exposure of sodium benzoate at a dose (100 mg/kg) in rats resulted in decreased bodyweight. Similar results have been shown by Fujitani (1993) for 10 days of intake 2.40% of SB in the diet in rats and mice (43). But, contrary to the study of Kehinde *et al*. (2018), who observed no significant change in body weight gain of SB-treated group (4). Then, the administration of* Atriplex* post sodium benzoate intoxication restored the weight of the body as normal, which can indicate the beneficial effect of Ah against SB as evidenced macroscopically. 

Creatinine and urea are waste products of protein metabolism that need to be excreted by the kidney (44); therefore, the increased levels of serum urea and creatinine in this study reflected the functional damage of the kidney. This result was associated with a significant decrease in serum protein and an increase in serum LDH. However, there were no significant changes in serum glucose, uric acid, and albumin concentration in the SB group compared with the control group. On the other hand, the aqueous extract of *A. halimus* treatment in both types decreased the raised serum creatinine, urea, and LDH levels following by increased protein levels. This means that *Atriplex* has maintained kidney function, which can be related to its bioactive compounds.

Oxidative stress has a critical role in the pathophysiology of several kidney diseases. It promotes renal injury via damage to molecular components of the kidney through the activities of intra- and extracellular reactive oxygen radicals (45). Our results clearly indicated that sodium benzoate exposure induced the impairment in the antioxidant defense system associated with the accumulation of oxidative free radicals in the kidney tissue. The reason is that the critical antioxidant indices GST, CAT, and GSH levels decreased and lipid peroxidation marker MDA increased after SB exposure. Kehinde *et al*. (2018) found that SB induced increases in plasma MDA (4). In fact, Lipid peroxidation is one of the primary effects induced by oxidative stress (46, 47). It is a chain reaction created by free radicals leading to deteriorative changes in the cell’s structure and function (48). Hence, the increased MDA level in this study reflects the oxidative stress status induced by SB in kidney tissue. The possible mechanism that the SB induced oxidative stress may be due to their important metabolite hippuric acid (HA), which is cleared by the kidney and excreted in the urine. Indeed, according to Kim *et al.* (2011), there are significant associations between HA levels and oxidative stress markers (49). They found that HA ratios affected MDA ratios significantly and found significant changes of 23 gene expression by HA; this gene may be associated with oxidative stress. Then, Nissar *et al*. (2017) showed a significant relationship between urinary HA and genetic polymorphisms of GSTs. Glutathione S-transferases (GSTs) are a multigene family of detoxifying enzymes, protecting cells against xenobiotics chemicals and reactive oxygen species (50).

In addition, the CAT enzyme is the primary line of defense against oxidative injury (51). The decreased GST and CAT activities are attributable to the reduced synthesis of these enzymes. Lastly, GSH is a major intracellular antioxidant molecule; it is the first line of defense against free radical attacks as a non-enzymatic antioxidant (52, 53). In this study, depletion of GSH after SB exposure may reflect its consumption by the overproduction of ROS and its conjugation with SB or their metabolites. Treatment with aqueous extract of* A. halimus*, mainly in curative mode, dramatically diminished MDA level and increased GSH, CAT and GST level as compared with SB group. Thus, these results strongly demonstrate the potential antioxidant activity and a high ROS scavenging capacity of* A. halimus*. This effect can be attributed to its flavonoid and phenolic content as these components possess potent antioxidant and anti-lipoperoxidative activities (54). ‬

Histopathological examination of kidney tissue from the sodium benzoate group confirmed the biochemical results and showed clear signs of nephrotoxicity. Microscopic observation of SB section revealed severe glomerular and tubular alteration, glomeruli atrophy, nephrocellular necrosis vascular congestion, inflammatory cell infiltration, vacuolization of tubular cells, tubular dilatation. However, *Atriplex* extract improved the renal histopathological alterations with the potential restoration of glomeruli and tubules structure near to normal status in both modes of treatment. This effect of the extract may be related to the presence of anti-inflammatory and antioxidant phytocomponents in this plant.

**Table 1:** Phytochemical composition of aqueous extracts of *Atriplex halimus*

**Phytochemical**	***Atriplex halimus***
Flavonoids	++++
Tannins	++++
Terpenoids	++++
Alkaloids	+
Saponins	++++
Anthocyanins	++++
Cardiac glycosids	++
Mucilage	++
Coumarins	++
Carotenoids	−

+ = presence of phytochemicals;

− = absence of phytochemicals;

++++ = high concentration;

++ = moderate concentration.

**Table 2:** Total phenolic and flavonoids content

**Compounds**	***Atriplex halimus***
Total phenolic (mg GAE eq/g dry wt)	17.183 ± 0.067
Flavonoids (mg Q eq/g dry wt)	4.024 ± 0.0013

**Table 3:** Initial body weight, final body weight, body weight gain and relative kidney weight in control and experimental groups (n = 6).

**Parameters**	**Control**	**Ah**	**SB**	**SB+AhC**	**AhP+SB**
Initial body weight (g)	221.17 ± 7.73	233 ± 4.71	204.7 ± 15.2	229 ± 14.5	215.8 ± 14.8
Final body weight (g)	299.17 ± 6.6	308.5 ± 13.6	262.3 ± 23.1^*^	286 ± 13.3^**^	293 ± 18.1^b^
Body weight gain (g/day)	0.74 ± 0.10	0.719 ± 0.064	0.55 ± 0.049^*^	0.54 ± 0.032^**^	0.73 ± 0.05^b^
Absolute kidney weight (g)	1.43 ± 0.06	1.49 ± 0.051	1.22 ± 0.14	1.37 ± 0.083	1.38 ± 0.072
Relative kidney weight (g/100 g bw)	0.47 ± 0.019	0.48 ± 0.014	0.46 ± 0.02	0.47 ± 0.017	0.47 ± 0.015

^*^ *p *< 0.05, ^**^p < 0.01: significantly different from control group.

^b^ *p *< 0.01: significantly different from SB group.

Values are mean ± SEM, n = number of observations.

**Table 4:** Mean blood glucose levels and blood biochemical values in the control and experimental groups (n = 6).

**Parameters**	**Control**	**Ah**	**SB**	**SB+AhC**	**AhP+SB**
Blood glucose (g/L)	1.026 ± 0.059	0.93 ± 0.039	1.038 ± 0.05	1.024 ± 0.045	1.09 ± 0.07
Serum urea (g/L)	0.27 ± 0.017	0.28 ± 0.009	0.29 ± 0.005^*^	0.27 ± 0.017	0.15 ± 0.023^**c^
Serum creatinine (mg/L)	7.45 ± 0.16	7.85 ± 0.20	9.56 ± 0.44^**^	7.68 ± 0.26^a^	7.52 ± 0.45^a^
Blood ratio urea/creatinine	37.58 ± 3.85	35.66 ± 2.84	30.33 ± 4.12^*^	35.15 ± 2.93^a^	23.27 ± 1.74^***b^
Serum uric acid (mg/L)	19.67 ± 1.33	14.59 ± 0.55^***^	19.90 ± 3.45	20.73 ± 1.75	15.90 ± 0.93^*a^
Serum albumin (g/L)	31.83 ± 1.33	39.33 ± 1.41	29.40 ± 0.24	32.25 ± 0.94	39.50 ± 1.32
Serum protein (g/L)	72.33 ± 2.47	83.83 ± 3.42^*^	60.50 ± 2.96^**^	66.25 ± 2.95^a^	70.60 ± 3.50^a^
Serum LDH (U/L)	3166 ± 149	2227 ± 135^**^	3554 ± 111^**^	3131 ± 97^a^	2965 ± 165^a^

^*^ *p* < 0.05, ^***^p < 0.001, ^**^p < 0.01: significantly different from control group.

^a^ *p* < 0.05, ^b^*p* < 0.01, ^c^*p* < 0.001: significantly different from SB group.

Values are mean ± SEM, n = number of observations.

**Table 5:** Malondialdehyde, Reduced Glutathione level, Glutathione-S-transferase, and Catalase activities in the kidney of control and experimental groups (n = 6)

**Parameters**	**Control**	**Ah**	**SB**	**SB+AhC**	**AhP+SB**
MDA (nmol/mg prot)	3.78 ± 0.33	3.98 ± 0.18	4.87 ± 0.325^*^	2.29 ± 0.18^**c^	3.86 ± 0.20^a^
GSH (nmol/g tissue)	2.97 ± 0.21	2.42 ± 0.31	1.86 ± 0.19^*^	2.43 ± 0.23^a^	2.22 ± 0.13^*a^
GST (nmol/min/g tissue)	5.96 ± 0.49	6.26 ± 0.65	4.07 ± 0.45^**^	6.37 ± 0.59^a^	4.95 ± 0.49
CAT (U/g tissue)	1.16 ± 0.044	1.21 ± 0.25	0.86 ± 0.078^*^	1.26 ± 0.12^b^	0.88 ± 0.098^*^

^*^ *p *< 0.05, ^**^p < 0.01: significantly different from control group.

^a^ *p *< 0.01, ^b^*p *< 0.01, ^c^*p *< 0.001: significantly different from SB group.

Values are mean ± SEM, n = number of observations.

**Table 6:** Correlation coefficients and the significant levels of different kidney marker components in rats

**Ratio Ur/Cr**		**Urea**		**Creatinine**		**Component ** ***vs.***
**P**	**R**	**P**	**R**	**P**	**R**	
0.39	-0.30	0.92	0.056	0.04	0.64	Kidney MDA
0.05	0.63	0.96	0.76	0.01	-0. 76	Kidney GSH
0.034	0.67	0.73	0.207	0.012	-0.75	Kidney GST
0.003	0.82	0.42	0.46	0.098	-0.52	Kidney CAT

**Table 7:** Semi-quantitative recording of architectural damage on histopathological analysis of the kidney of control and treated rats

**Parameters**	**Control**	**Ah**	**SB**	**SB+AhC**	**AhP+SB**
Glomerular atrophy	^_^	^_^	^++^	^_^	^_^
Inflammatory infiltration	^_^	^_^	^+++^	^+^	^_^
Tubular and glomerular necrosis	^_^	^_^	^+++^	^-^	+
Dilatation and tabular vacuolation	^_^	^_^	^+++^	^_^	^_^

## Conclusion

The present study demonstrates that *Atriplex halimus *can reduce (or prevent) several toxicities induced by sodium benzoate in kidneys. This protective effect might be attributed to its antioxidant and free radical scavenging properties. Thus, *Atriplex halimus* can be used as a nephroprotective agent and protect the kidney from SB-induced injury.
